# Nonrecurrent Laryngeal Nerve in the Era of Intraoperative Nerve Monitoring

**DOI:** 10.1155/2016/1606029

**Published:** 2016-10-13

**Authors:** Emin Gurleyik, Gunay Gurleyik

**Affiliations:** ^1^Department of Surgery, Faculty of Medicine, Duzce University, Duzce, Turkey; ^2^Department of Surgery, Haydarpasa Numune Research Hospital, Istanbul, Turkey

## Abstract

Nonrecurrent laryngeal nerve (non-RLN) is an anatomical variation increasing the risk of vocal cord palsy. Prediction and early identification of non-RLN may minimize such a risk of injury. This study assessed the effect of intraoperative neuromonitoring (IONM) on the detection of non-RLN. A total of 462 (236 right) nerves in 272 patients were identified and totally exposed, and all intraoperative steps of IONM were sequentially applied on the vagus nerve (VN) and RLN. Right predissection VN stimulation at a distal point did not create a sound signal in three cases (3/236; 1.27%). Proximal dissection of the right VN under IONM guidance established a proximal point, creating a positive signal. The separation point of non-RLN from VN was discovered in all three patients. Non-RLNs were exposed from separation to laryngeal entry. Positive IONM signals were obtained after resection of thyroid lobes, and postoperative period was uneventful in patients with non-RLN. Absence of distal VN signal is a precise predictor of the non-RLN. IONM-guided proximal dissection of the right VN leads to identification of the non-RLN. The prediction of non-RLN by the absence of the VN signal at an early stage of surgery may prevent or minimize the risk of nerve injury.

## 1. Introduction

Identification and full exposure of the recurrent laryngeal nerve (RLN) are mandatory for safer thyroid surgery. Knowledge of the anatomy of the RLN, including its anatomical variations, good surgical skill, and experience are required for preserving anatomical integrity and motor activity of the nerve. Despite the rarity of the non-RLN, it is accepted as an important anatomical variation increasing the risk for vocal cord palsy. To minimize the risk of the nonrecurrent course, several studies have been conducted to predict the presence of non-RLN preoperatively. Based on the association with vascular abnormality, researchers have attempted to identify this vascular variation by radiological methods to predict the associated non-RLN. Some imaging methods were found useful for the detection of vascular abnormality that can accurately predict the existence of non-RLN [1–5]. Safer thyroid surgery depends on the preservation of the motor activity of the RLN; thus, it is extremely important to determine the integrity of the motor function. Intraoperative neuromonitoring (IONM) is a widely accepted method to evaluate the motor activity of the RLN during thyroid surgery [6–8]. We hypothesize that IONM provides additional findings for the early detection of non-RLN during thyroid surgery.

In this article, we aim to present our results of IONM in our cases of non-RLN to determine electrophysiological parameters for the early and safe detection of the nonrecurrent nerve.

## 2. Materials and Methods

We conducted this prospective study on IONM in thyroid surgery between January 2013 and December 2015. All steps of IONM were properly performed on 272 primary thyroid surgeries (190 total thyroidectomies, 46 right hemithyroidectomies, and 36 left hemithyroidectomies). 462 (236 right and 226 left) RLNs were anatomically identified and exposed until the point of laryngeal entry during the thyroid surgeries. Functional identification of the vagus and laryngeal nerves was performed by means of IONM.

### 2.1. Intraoperative Neuromonitoring of the Vagus and the RLNs

IONM was performed using the Nerve Integrity Monitor (NIM-Response 3.0 System; Medtronic Xomed, Jacksonville, FL, USA). The setup of the device was 1 mA as the stimulation intensity and 100 *μ*V as the amplitude threshold. The nerves were directly stimulated after their visual identification. Standard IONM was performed as a four-step procedure, as follows.

*V1 (Predissection Vagus Nerve (VN) Stimulation)*. Lateral thyroid lobe was partially mobilized after ligation of middle thyroid vein. After medial traction of the thyroid lobe, the VN was identified in the neurovascular bundle. Carotid sheet was incised and the nerve was found behind the carotid artery and the jugular vein. The stimulator probe was touching the VN. If any, the wave amplitude was recorded in *μ*V.

*R1 (Predissection RLN Stimulation)*. RLN was stimulated when first identified near the inferior thyroid artery, and the amplitude was also recorded in *μ*V.

After visual and electrophysiological identification, the cervical part of the RLN was carefully dissected and fully exposed until the laryngeal entry.

*R2 (Postdissection RLN Stimulation)*. The RLN was stimulated after complete dissection of the lateral thyroid lobe, and the amplitude was recorded in *μ*V.

*V2 (Postdissection VN Stimulation)*. The VN was stimulated after complete dissection of the lateral thyroid lobe, and the amplitude was recorded in *μ*V.

Laryngoscopy was pre- and postoperatively performed to check vocal cord mobility.

### 2.2. RLN Dissection Technique

After freeing and medially mobilizing the bilateral lobes of the thyroid gland, the inferior thyroid arteries were identified and isolated. Using a conventional lateral approach, the RLN was identified below the artery. R1 stimulation and signal were first recorded following identification of the nerve. The RLN was fully isolated and exposed on both sides until the laryngeal entry point.

### 2.3. Non-RLN Dissection Technique

If early nerve monitoring revealed the presence of non-RLN, carotid sheet incision was extended toward the cephalic direction in order to access and isolate proximal VN. The nerve was proximally followed under guidance of IONM with serial electrophysiological stimulation to identify the separation point of the inferior laryngeal nerve. Surgical anatomy and integrity of the laryngeal nerves were established using careful surgical exposure throughout the cervical course. Motor activity and integrity of the neural structures were determined by IONM.

## 3. Results

 In the last 3 years, all steps of IONM could be properly performed in 272 primary thyroidectomy cases. The cervical parts of 462 (236 right and 226 left) RLNs were identified and fully exposed.

**Figure 1:**
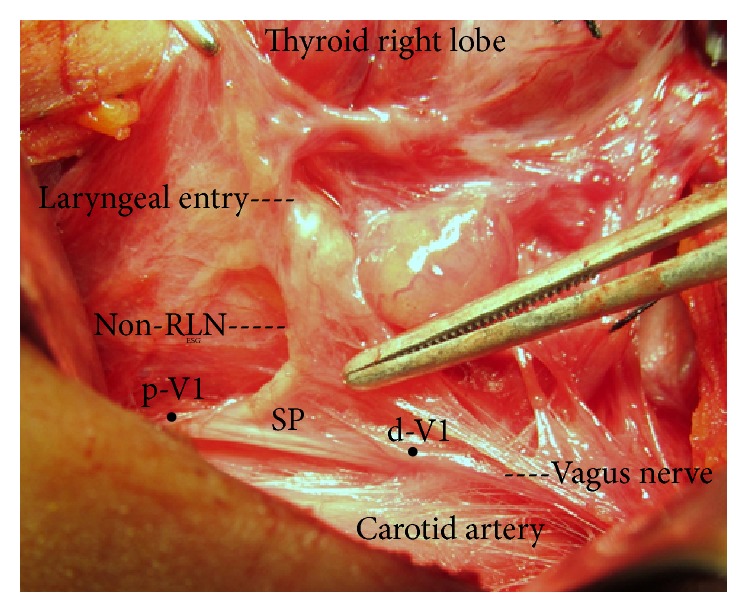
Full exposure of right nonrecurrent laryngeal nerve (non-RLN) from separation point (SP) on the vagus nerve to laryngeal entry. Electrophysiological signal is negative at distal stimulation point (d-V1) and positive at proximal stimulation point (p-V1) on the vagus nerve before separation of the inferior laryngeal nerve.

Right V1 stimulation created positive sound signal, and the wave amplitude was measured in all nerves, except in three cases (3/236; 1.27%) in which the V1 signals from the right VNs were negative at a distal contact point of the stimulator probe. Taking into account an anatomical variation of the RLN, upper poles of right lobes of the thyroid gland were carefully dissected and caudally mobilized in these three patients. Early dissection of the right vagus was performed before dissection of paratracheal region. Nerve dissection was performed in an upward direction along the right vagus under the guidance of IONM with serial applications of stimulator probe at a distance of 1 cm in each nerve segment. Positive signal from a proximal point of stimulation helped us to discover the separation point of the right inferior laryngeal nerve from the VN in all the three patients. These nerves arising from proximal vagus were fully exposed during the thyroid surgery. The nerves had a nonrecurrent course of approximately 3-4 cm until the laryngeal entry. Vagus signal was negative if stimulator probe contact point was located distal to the non-RLN separation and positive if it was located proximal to the separation (Figure 1).

Distal V1 signals from the right VNs were negative after initial vagus stimulation. Mean proximal vagus (p-V1) wave amplitude was recorded as 980 (range: 639–1350) *μ*V and mean R1 amplitude as 1227 (range: 612–2061) *μ*V in patients with right non-RLNs. The latencies of p-V1 were measured as 2.3, 3.3, and 3.6 milliseconds (mS) in non-RLN cases. Mean right V1 and R1 amplitudes were 803 (range: 306–1963) *μ*V and 996 (range: 324–3297) *μ*V, respectively, and latencies were between 4.8 and 6.7 mS in the remaining patients with the normal RLN.

Three patients with non-RLN were women. The diagnosis was papillary carcinoma in one and toxic multinodular goiter in two patients. We performed total thyroidectomy on these patients. Positive R2 and proximal V2 sound signals were obtained after full dissection of thyroid lobes. Mean right R2 and V2 amplitudes were recorded as 989 (range: 562–1570) *μ*V and 879 (range: 483–1325) *μ*V, respectively, after resection of thyroid lobes. Postoperative period was uneventful in three patients with non-RLN who were discharged on the second postoperative day. In our total series of patients, 2 cases (2/462; 0.43%) of unilateral RLN injury and vocal cord palsy have occurred as postoperative neural complication.

## 4. Discussion

Anatomical variations of the RLN, increasing the risk of injury to the nerve, threaten the safety of thyroid surgery [7, 9–11]. Despite the rarity, nonrecurrent course of the nerve deserves a special attention in order to minimize the risk of vocal cord palsy. Prediction of the presence of the non-RLN preoperatively or at the beginning of surgery can eventually minimize injury risk to the nerve. For the last 4 years, we have been using IONM in all patients undergoing thyroid and parathyroid surgery. If the IONM is properly performed, it seems as a useful adjunct to anatomical identification of the RLN. Electrophysiological stimulation of the vagus and laryngeal nerves theoretically helps to predict the existence of non-RLN at the early intraoperative period and may provide parameters to facilitate identification of non-RLN.

The incidence of non-RLN is very low and has been reported to be between 0.5 and 6% [2, 3, 10, 12–16]. The non-RLN has attracted more importance, interest, and attention than its very low incidence. Therefore, clinical and radiological studies were conducted to predict the presence of non-RLN preoperatively in order to minimize the risk of nonrecurrent course and prevent nerve injury due this anatomical variation [2, 3, 5]. Based on the association with the right aberrant subclavian artery with a retroesophageal course (arteria lusoria), thoracic computed tomography (CT) and ultrasonography have been reported to be useful for detecting the absence of brachiocephalic trunk and aberrant subclavian artery, which can accurately predict the existence of non-RLN [1–5]. In three previous studies, ultrasound was performed on 3634 patients who were scheduled for thyroid surgery, which preoperatively predicted non-RLN in 46 (1.3%) patients [2–4]. Neck CT was performed on 4257 patients that identified vascular anomaly and predicted the non-RLN in 22 (0.5%) patients [5, 12, 13, 17]. Are preoperative ultrasound and CT in all cases of thyroid surgery cost-effective, beneficial, and advantageous? It is difficult to positively answer this question in order to discover the non-RLN in 1%-2% of patients. Therefore, we believe that this low incidence does not support the use of imaging methods in all candidates of thyroid surgery to discover vascular anomaly and the non-RLN, especially in the era of IONM.

Preservation of anatomical integrity and motor activity of the nerve has paramount importance for a successful thyroid surgery. IONM is a widely accepted method for intraoperative assessment of neural motor function [6–8]. We propose routine use of nerve monitoring in all thyroidectomy cases as a useful adjunct to visual exposure of the RLN. Changes in physiology of the vagus and laryngeal nerve system determined by IONM were studied in our cases of non-RLN. Nerve monitoring begins with the first step of vagus stimulation (V1) at the level of lower third of the thyroid (distal cervical vagus). The absence of signal and electrophysiological wave amplitude at this level reveals a disorder of signal transmission by motor fibers of the inferior neural system to intrinsic laryngeal musculature. Taking into account proximal branching of the laryngeal nerve from the vagus as an anatomical variation, the absence of motor function after distal V1 stimulation reveals the presence of nonrecurrent course of the nerve. In our cases, the absence of electrical conductivity in the motor nerve after right V1 stimulation urged us to proximally dissect along the VN, with serial electrophysiological stimulations, until the separation point of the laryngeal nerve. A positive signal at the proximal point on the VN with serial stimulations helps in detecting the branching of the laryngeal nerve. Therefore, a negative right V1 signal at the distal point appeared as an early intraoperative indicator of the non-RLN. Chiang et al. [14] had published in 2012 their results of IONM in four cases of non-RLN. They have reported that non-RLNs without preoperative recognition were successfully detected at an early stage of surgery by IONM, with a negative response from lower vagal stimulation. Cai et al. [12] had preoperatively identified vascular variation by neck CT in 4 of 783 cases. They have reported that the separation point of the non-RLNs was identified and precisely localized with IONM. Gao et al. [13] had also identified 9 cases of non-RLNs based on vascular anomalies in neck CT scans of 1574 patients scheduled for thyroid surgery. All nine cases were also successfully detected at an early stage of surgery using IONM. A negative response from lower vagal stimulation indicated the occurrence of a non-RLN [13]. Donatini et al. [16] have reported that using a systematic IONM can increase detection of non-RLN and decrease the incidence of nerve palsy in cases of non-RLN. Kamani et al. [18] identified 10 right-sided non-RLNs by application of IONM. They reported that monitoring vagal stimulation at the defined distal and proximal points provides reliable verification of the presence of the non-RLN [18]. Signals derived from the VN were positive if derived proximal to and negative if derived distal to the branching of a non-RLN [15]. Our results on the three cases demonstrate the importance of IONM and especially right V1 stimulation and signal in predicting the presence of non-RLN at an early stage of thyroidectomy. Five previous reports based on IONM application in non-RLN cases also verify the role of negative V1 signal in enhancing the detection and safe identification of the non-RLN.

In IONM, the latency is defined as the time (as mS) between stimulation and peak of first evoked wave. Intraoperative initial vagus stimulation (V1) must create positive signal for calculation of latent period between vagus stimulation and first wave peak. In our patients with non-RLN, signal was negative after standard (distal) V1 stimulation. Therefore, in our study, absence of initial V1 signal appeared as the main and primary sign of presence of the non-RLN. Then we were able to calculate the vagal latency just after proximal stimulation of the vagus at a point proximal to separation of the laryngeal nerve. The latency was previously reported as a sign of non-RLN [19]. Brauckhoff et al. [19] have reported that latencies shorter than 3.5 mS after vagus nerve stimulation signify a nonrecurrent inferior laryngeal nerve before dissection. On the contrary, three years later, Brauckhoff et al. [20] have reported that latencies longer than 3.5 mS after vagus nerve stimulation do not exclude a nonrecurrent inferior laryngeal nerve.

IONM, as an adjunct to visual identification of the RLN during thyroid surgery, monitors proper transmission of motor signal via the vagus and laryngeal neural system to the intrinsic laryngeal musculature. IONM assistance is especially important in case of neural anatomical variations. The absence of early V1 signal at distal stimulation of the right VN indicates an interruption of transmission path secondary to early branching of inferior laryngeal nerve before the stimulation point. Anatomical and neurophysiological identification of the separation point of the inferior laryngeal nerve is accomplished by proximal dissection under the guidance of IONM. Therefore, verification of the presence of the non-RLN is precisely provided at an early stage of the surgery by IONM. Surgeons should be familiar with all the possible course variations in the RLN when IONM signals are absent with vagal stimulation.
